# Experiences of Patients with Breast Cancer Participating in a Prehabilitation Program: A Qualitative Study

**DOI:** 10.3390/jcm13133732

**Published:** 2024-06-26

**Authors:** Anabel Casanovas-Álvarez, Raquel Sebio-Garcia, Jaume Masià, Ester Mateo-Aguilar

**Affiliations:** 1 Department of Health Sciences TecnoCampus, Universitat Pompeu Fabra, 08302 Mataró, Spain; acasanovasa@tecnocampus.com (A.C.-Á.); emateo@tecnocampus.cat (E.M.-A.); 2 Research Group in Chronic Care and Health Innovation (GRACIS) TecnoCampus, Universitat Pompeu Fabra, 08302 Mataró, Spain; 3 Physical Therapy Department, Universitat Internacional de Catalunya, 08017 Barcelona, Spain; 4 Department of Physical Medicine and Rehabilitation, Hospital Clinic de Barcelona, 08036 Barcelona, Spain; 5 Department of Plastic and Reconstructive Surgery, Hospital de la Santa Creu I Sant Pau, Universitat Autònoma de Barcelona, 08025 Barcelona, Spain; jmasia@santpau.cat

**Keywords:** prehabilitation, breast cancer, qualitative research, barriers, facilitators, peer-support, multidisciplinary care

## Abstract

**Background:** Breast cancer (BC) is the most prevalent cancer diagnosis among women worldwide. Several randomized controlled trials and systematic reviews have shown the benefits of exercise before, during, and after cancer treatment to manage side effects related to cancer and its therapies. However, these are poorly implemented across the disease-span, specifically, during the preoperative setting. **Methods:** Patients diagnosed with BC and participating in a randomized controlled trial on the effects of a prehabilitation program based on Nordic walking, muscle strengthening, and therapeutic education were invited to participate in this qualitative substudy. Two groups of eight patients each were recorded, transcript and analyzed using a specialized software (Atlas-Ti^®^, version 24). **Results:** During the axial codification phase, 22 unique codes and 6 main themes were identified related to their experience with the program, namely, (1) information received prior to participating; (2) motivation to participate; (3) barriers; (4) facilitators; (5) perceived degree of support from healthcare workers as well as peers; and (6) satisfaction with the characteristics of the prehabilitation program. **Conclusions:** Patients interviewed showed great interest in prehabilitation as a way to prepare both physically and mentally for surgery. In order to implement these interventions, healthcare systems need to acknowledge barriers and facilitators as well as the need for these programs to be supervised and monitored to avoid adverse events.

## 1. Introduction

Breast cancer (BC) is the most common female tumor and the leading cause of death in women [1]. Thanks to early diagnosis and improvements in treatment modalities, the prognosis and survival rate of women with breast cancer have improved considerably worldwide [2] and nowadays approximately 88% of patients with BC will survive 5 years or more. However, this increase in survival rates has led to an increase in the number of patients facing disease-related issues requiring ongoing care after cancer treatment [3]. In particular, women with BC face different problems and needs in several dimensions (physical, psychological, and social) [4]. For instance, in 2023, a systematic review on the needs of patients living beyond BC showed that the majority of them had psychological and information needs that were not covered [5].

Several studies have investigated support needs to cope with problems such as pain, fatigue, neuropathies, cognitive impairment, body image concerns, osteoporosis, traumatic stress, fear of recurrence, sexual problems, sleep disturbances, and financial distress in patients with BC [6,7,8]. It is well-known that these factors contribute to slow recovery from stress and hinder return to work and normal social activities [5]. To restore functionality in these patients, an accumulating body of evidence over the last few decades has shown the potential benefits of physical training and structured exercise during and after cancer treatment [9]. Given the variety of side effects that these patients may experience, physical training should be combined with psychological, physical, and social care among others [10]. In addition, it has been recently shown that the earlier these interventions start, the better the outcome.

Oncological prehabilitation comprises a multimodal approach to patient care encompassing preoperative physical exercise, psychological support, and nutritional optimization. The goal of these programs is to improve the individual’s functional ability to withstand the stressors of surgery, thereby potentially accelerating postoperative recovery and improving outcomes [11,12]. In particular, physical activity has been shown to be associated with reduced fatigue and improved health-related quality of life and physical functioning in women with BC [13], as well as generate emotional benefits and foster peer-support during the sessions [14]. The preoperative period is known to represent a teachable moment in healthcare as individuals may be more receptive to integrating behavioral changes into their lifestyle [15]. However, without good adherence, future prehabilitation interventions are unlikely to be effective in improving outcomes [16]. Despite the potential benefits of prehabilitation, there are barriers to successfully implement these programs in clinical practice, both patient- and system-related [11,17,18]. As for the patients’ willingness to participate in these programs, aspects such as current physical functioning and co-morbidities, social support, or lack of time due to medical appointments or other commitments have been identified in the literature. In addition, patient acceptance of the intervention is essential for any prehabilitation program to be successful; thus, it is crucial when designing prehabilitation interventions to take into account patients’ preferences as well as potential facilitators and barriers to these programs [19]. The important factor in providing quality care is to identify and meet the needs of this group of patients in order to tailor healthcare services to the needs, preferences, and concerns of these patients by linking data from quantitative studies to help facilitate better cancer care [20].

Therefore, the aim of this qualitative study was to analyze the perceptions and experiences of patients with BC who participated in a prehabilitation program (PREOptimize) [21] in order to improve future implementations of these much-needed programs for patients with breast cancer.

## 2. Materials and Methods

### 2.1. Design

A qualitative descriptive phenomenological study was conducted from the perspective of Gadamer’s hermeneutic phenomenology [22]. This approach aimed to achieve results that responded to the lived experiences of the participants aligning with the study’s objective.

This qualitative study followed the randomized clinical trial PREOptimize, focusing on the impact of a prehabilitation program consisting of Nordic walking training, muscle strengthening exercises and therapeutic education compared to standard of care in patients with BC undergoing neoadjuvant treatment and awaiting surgery. The interviews were conducted after the main study was closed, that is, when all participants had recovered from surgery. This substudy was approved as part of the main research by the local ethics committee (IISBP-PRO-2021-10). Patients signed specific informed consent to be recorded and included in the qualitative analysis.

The reporting of findings adhered to the consolidated COREQ criteria [23].

### 2.2. Context and Participants

The study was conducted in the oncology unit of the Hospital de Santa Creu i Sant Pau, located in the province of Barcelona, Catalonia, Spain. Women with BC who were previously randomized to a prehabilitation program as described before were intentionally recruited for two focus groups. To ensure confidentiality, participants were identified using the format P- and a random number ranging from 1 to 18. The inclusion criteria included patients with BC participating in the PREOptimize trial during neoadjuvant treatment and voluntary consent to participate in the focus groups. Exclusion criteria considered post-surgery health conditions that would prevent the focus group from being executed.

Thirty-two participants from the clinical trial intervention group were invited via telephone and email to attend a face-to-face briefing meeting at the referral hospital. Twenty-one women attended the meeting and were informed about the voluntary participation in the focus groups. The other eleven women who did not attend the briefing meeting did not respond to the subsequent telephone calls. Of these 21 participants, 16 women ultimately participated in the focus group. The five interested participants were unable to attend due to various reasons such as childcare responsibilities, holiday commitments, and health problems.

### 2.3. Prehabilitation Program

The prehabilitation program started on the fourth month of chemotherapy and lasted until surgery. Full details of the intervention can be found in the protocol [21]. In brief, each session of 75 min was structured as follows: (i) a 10 min general warm up; (ii) a 50 min main part which consisted of 5 min of technique instruction and two blocks of 10–15 min of NW interspersed with two blocks of 10 min body-weight strengthening exercises, and (iii) a 15 min cool-down.

### 2.4. Data Collection

The focus groups were conducted in the interaction room of the Research Institute of the Hospital de la Santa Creu i Sant Pau, a familiar environment to the participants. All women were informed on the study’s purpose before they gave their signed consent. A first focus group was conducted, and once the results were analyzed, a second focus group followed to ensure saturation of the sample [24]. The focus groups were recorded simultaneously with two Zoom and H4n model digital recorders and lasted 75 and 70 min, respectively.

Between May and July 2023, two researchers participated in data collection. One researcher was dedicated to observing the focus groups and noting details such as real-time occurrences, various interruptions and/or external noises, gestures, and/or prolonged silences. For this purpose, a digital stopwatch was used discreetly, visible only to R.S.-G. for note-taking purposes. Another researcher was responsible for moderating the focus groups using a script of semi-structured questions. The script comprised an initial introduction, five blocks of semi-structured questions, and a final phase where the participants had the opportunity to freely express additional insights. This script addressed the perception and opinions of women with breast cancer regarding the current prehabilitation design, barriers, and facilitators, as well as the support and follow-up of the multidisciplinary team throughout their disease process. The focus groups concluded with a discussion on the participant´s satisfaction with the prehabilitation program, during which they were able to contribute ideas for improvements for future prehabilitation designs and express any previously unaddressed concerns. The participants were acknowledged for their contribution to the project and informed that they could be contacted for future clarifications post-transcription and after the analysis for a better understanding of the discussions.

### 2.5. Data Analysis

Thematic analysis was used to identify, analyze, and report patterns (themes) within the data following a structured procedure of six phases [25]: (a) Familiarization with the data involved transcribing, reading, and re-reading the data, along with jotting down initial ideas; (b) initial notable features of the data or codes were generated; (c) codes were collated and examined for potential themes; (d) themes were reviewed and a thematic map of the analysis was generated; (e) themes were defined and named, and their specifics refined; and (f) the analysis report was produced. To ensure the quality of the data, the information was contrasted by comparing multiple information sources, techniques, and groups of informants. Additionally, it was also reviewed by three analysts with different academic backgrounds and good knowledge of the context.

The focus group recordings were transcribed by the principal investigator and a third author after each session, taking into account the observations noted by the observer. Subsequently, content validation by the participants themselves was performed to maintain their reliability. The data collected from the focus groups were subjected to a rigorous systematic and comparative analysis organized into conceptual themes in relation to the study´s objective. This analysis was supported by Atlas.Ti^®^ version 24 software, and all researchers participated in the different stages of the data analysis process. Initial codes were formed from reading the quotes, forming units of meaning, which further contributed to the identification and formation of the main themes of the study.

## 3. Results

From the 32 patients invited to participate in the focus groups, 16 women between 37 to 68 years of age agreed to attend and were distributed in two focus groups, each comprising eight participants. The main characteristics of the patients included in the analyses are shown in Supplementary Materials (Tables S1 and S2).

**Figure 1:**
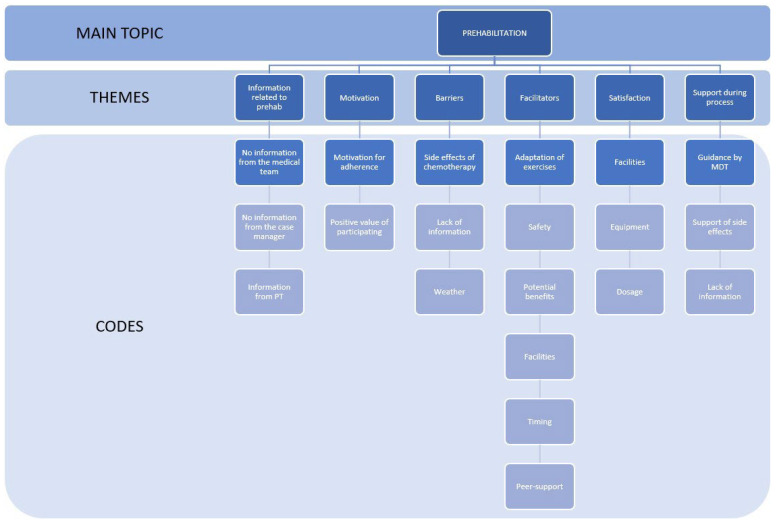
Summary of the identified themes and their respective codes from the two focus groups.

The opinions and perceptions expressed by women with breast cancer who participated in the PREOptimize prehabilitation program were grouped into six main topics and 22 codes associated with each topic. The core category that arises from the selective coding process is “Facilitators for participation and adherence to the prehabilitation program”. Six thematic axes emerged from the qualitative analysis of the participants’ free descriptions of the PREOptimize program. Among these axes, multiple aspects such as the following were described: (i) information received prior to the prehabilitation study; (ii) motivation and positive value of the choice to participate in the prehabilitation program; (iii) barriers to participation and adherence to the exercise regimen; (iv) facilitators for participation and adherence to the prehabilitation program; (v) degree of satisfaction with the prehabilitation program; and (vi) guidance and support by the multidisciplinary team offered to the patients during the treatment for BC. Figure 1 provides the results according to the topics and codes obtained for better understanding.

In addition, on the following pages, each topic is described in detail along with their corresponding codes and the most pertinent quotes.

### 3.1. Information Prior to the Prehabilitation Study

The participants refer to this point in terms of the information received from several specialists about the prehabilitation study in which they could participate.

#### 3.1.1. Lack of Information from the Medical Team

Regarding the information received by the participants so they could choose to participate in the PREOptimize prehabilitation program, multiple answers were obtained. On one side, most of the patients stated that they were not informed about the option of being able to participate in a prehabilitation study by the medical team or other members of the multidisciplinary team.

*“Zero information”.*

(ID7)

*“The medical team did not know about this prehabilitation intervention”.*

(ID10)

#### 3.1.2. Lack of Information from the Case Manager

Some other patients reported that they were encouraged to participate by the nursing case manager, who recommended them to participate in the study because of its benefits, but without receiving any detailed information about the program.

*“I was encouraged to participate by the nurse in charge of oncology. She told me that it would be good for me, without further explanation”.*

(ID8)

#### 3.1.3. Information Received from the Physical Therapist

Another group of women did feel better informed due to the first contact being made directly by the physiotherapist involved in the prehabilitation program. They were informed in detail about what the program consisted of and were given the dates and schedule, in addition to being able to ask any questions they had.

*“We learnt what Nordic walking was when the physiotherapist explained it”.*

(ID6)

### 3.2. Motivation for Adherence to the Prehabilitation Program and Positive Value of the Choice to Participate

This topic refers to the reasons why a participant may or may not be motivated to participate in the prehabilitation program. It describes the aspects perceived by participants that facilitate motivation and the reasons that generated positive value to the choice to participate.

#### 3.2.1. Motivation for Adherence to the Prehabilitation Program

The patients explained that they were unaware of the benefits that prehabilitation could have on managing side effects from chemotherapy in addition to other benefits on their physical, psychological, and social wellbeing. Furthermore, they acknowledged that being aware of that information might have had increased their motivation to participate in the study. They also reported that the greatest motivation for joining the program was to be guided and supervised by a physiotherapist specializing in oncology and to share those difficult moments by exercising outdoors (outside the hospital grounds) with people in the same situation where they could share and exchange experiences.

*“My motivation was to feel useful, to get out of the house. Otherwise, I wouldn’t go out”.*

(ID2)

*“The physiotherapist called me and just with her voice she motivated me”.*

(ID8)

The patients also acknowledged that although medical prescription was the oncologist’s first and most important role, they should also include the discussion of other topics such as the benefits of exercise to manage side effects from treatment, as this would enhance the motivation for participation.

*“It is up to oncologists to promote exercise and prescribe exercise to patients”.*

(ID15)

*“The biggest motivation is to feel the benefits”.*

(ID16)

#### 3.2.2. Positive Value of the Choice to Participate

This topic refers to the reasons why participants found positive value in participating in this program. In general, once all the women started the prehabilitation sessions, they reported that they went from feeling like fragile women undergoing chemotherapy to strong women doing high-intensity exercises.

*“I was quite shocked because they were high-intensity exercises. But I was very grateful, as I was at home and did nothing”.*

(ID10)

*“When I arrived and saw all those women like me, I got excited and said ‘This is my place’”.*

(ID14)

Thanks to the therapeutic education part of the program, they also understood that the physical condition with which one undergoes surgery is essential and had to be optimized.

*“Physical exercise should be part of the treatment. It’s not an option, it’s a must”.*

(ID13)

*“These prehabilitation programs are basic for anyone who is in a process like this”. *

(ID8)

From the very first sessions, all the participants began to report feeling both the physical and emotional benefits of the exercise supervised by a physiotherapist. Moreover, the fact that they were all together, in a group, with the same disease, at the same stage, and with different concerns, made them empathize with one another quickly. They felt understood and in a safe space of respect and confidence. These aspects were what really made them feel the positive value of participating.

*“I immediately saw a group of people in the same situation as me. I mean, it was the bald group, and we were all in the same place”.*

(ID11)

*“As an anecdote, I can say that it was the first day I took off my headscarf, and it was the first time I did it in public”.*

(ID16)

The type of supervision provided by the physiotherapist and the organization of the sessions were aspects that the participants also reported, which made them see the positive value in continuing with the sessions, reassuring them that they had made a good choice by participating.

*“The sessions were very well organized and supervised”.*

(ID5)

### 3.3. Barriers to Participation and Adherence to the Prehabilitation Program

This topic represents the challenges that some participants faced regarding participation and adherence during the prehabilitation program. They referred to aspects such as the side effects they experienced during their chemotherapy treatment and the lack of information and medical referral to prehabilitation, as well as the adverse weather conditions that could occur from time to time.

#### 3.3.1. Side Effects of Chemotherapy

One of the main barriers to participation in prehabilitation is the toxicity caused by the chemotherapy and its many side effects, such as decreased immune system function, tiredness and weakness, dizziness, neuropathies, muscle pains, etc.

*“If I was low on defenses it was difficult for me to go to the sessions”.*

(ID15)

*“Yes, occasionally, when you felt under the weather, you did get a bit dizzy”. *

(ID6)

#### 3.3.2. Lack of Information and Prescription for Prehabilitation

Participants also highlighted the lack of information and exercise prescription by the oncology service and the multidisciplinary team in general.

*“The oncologist knew that prehabilitation was a thing, but when I attended my check-ups he didn’t comment on it”.*

(ID16)

They believed that exercise should be part of anti-cancer therapy and that the medical team should engage patients in different actions that can have a positive impact on their process, instead of focusing so much on what they will not be able to do.

*“Physical exercise should be part of the treatment. It is not an option, it is a must”.*

(ID13)

The participants agreed that the whole medical team should be aware of prehabilitation programs. Any healthcare worker involved in the patient’s process—not only the nurse case manager or the physiotherapist as stated in this study—could then refer patients to such interventions.

*“For future reference, the medical team should inform and recommend participation in prehabilitation”.*

(ID13)

The participants stated, on several occasions, that the medical team—more specifically, the oncologists—should be the main specialists referring patients to these prehabilitation programs based on physical exercise. This should not be just an option but a main part of the treatment alongside surgery, chemotherapy, radiotherapy, hormone treatment, and other equally important treatments such as psychology and nutrition.

*“You should have professionals such as a psychologist, a physiotherapist, a nutritionist”.*

(ID6)

#### 3.3.3. Adverse Weather Conditions

Another factor that participants highlighted as barriers was the weather conditions. Being an outdoor activity, weather conditions such as rain, cold, wind, or excessive heat were also seen as barriers to adhering to the program. There was no specific location for these sessions to take place during adverse weather; thus, participants mentioned they would have appreciated, for example, a covered gym or a similar location. In addition, a participant added that some participants had to wear a face-mask due to COVID-19, and it was especially difficult for them to breathe during the exercise on very warm days.

*“On days when it was very windy or raining, the physiotherapist had to find a gym to do the session there”.*

(ID10)

### 3.4. Facilitators for Participation and Adherence to the Prehabilitation Program

The participants considered multiple aspects on the facilitators of the prehabilitation program that made participation and adherence easier for them. The codes that constitute this topic are as follows: (i) the professionalism and follow-up by the physiotherapist; (ii) the safety while taking part in an exercise regimen which was supervised by a physiotherapist; (iii) the individualization of exercises during the program, according to the patient’s status; (iv) the accessibility to the facilities where the program was carried out as well as frequency and timing; and (v) the mutual encouragement from all the members of the group to carry on with the sessions, which all participants highlighted.

#### 3.4.1. Professionalism and Follow-Up of the Physiotherapist

In this case, all participants described the professionalism and follow-up of the physiotherapist as one of the main facilitators for adhering to the program. They positively highlighted the therapeutic education that was provided and the diverse and structured exercise sessions they felt were necessary for their situation.

*“We have never met any other professional who has been so close, and I think that’s been part of the success”.*

(ID2)

*“The follow-up was spectacular. Very custom-made, very human”.*

(ID6)

#### 3.4.2. Adaptation of Exercises

The exercises were adapted to each participant according to their fluctuating conditions during chemotherapy. Any necessary exercise adaptations were designed for each patient depending on how they felt each day, in terms of the intensity and type of side effects reported. This ensured that the participants did not feel frustrated. The effort tolerance limit was respected so they were able to finish all sessions in complete safety.

*“Some of us felt bad […] We slowed down and the physiotherapist guided and controlled us”.*

(ID11)

#### 3.4.3. Safety of the Exercise Intervention

Custom-made exercise instruction tailored to each participant’s physical and mental condition, continuous supervision throughout the prehabilitation program, progression in terms of intensity, and the absence of injuries or other adverse effects caused by exercise made the participants feel safe and confident during the course of their exercise sessions.

*“Even if the level of exercise was high, we knew that nothing would happen to us”.*

(ID16)

#### 3.4.4. Benefits of the Intervention

Participants remembered their participation as being a very positive and enriching experience. The physical, psychological, and social benefits felt by participants throughout this prehabilitation program have been the main facilitators for adhering to the program. Participants reported that they felt many positive effects on their physical and emotional state, and some improvement in side effects such as fatigue, loss of muscle mass, alopecia stigmas, and overall mood. Moreover, they reported benefits such as being able to “let go” and talk about fears and feeling capable of much more, as strong, non-frail women.

*“I, as a patient, can verify that it has been physically and emotionally good”.*

(ID15)

*“The plastic surgeon told me that it was noticeable what I had done with the Nordic walking because the tissue was more elastic”.*

(ID11)

#### 3.4.5. Facilities

It was also important for the participants that the facilities were accessible. As the hospital is the referral one for the area, most of the patients could walk or take the metro to travel there. Spending so much time at medical appointments and with multiple follow-up tests at the hospital, the participants appreciated the fact that the prehabilitation would take place in the open air.

*“I was very grateful that it was outdoors”.*

(ID13)

*“I was coming by metro and my stop was right at the hospital”.*

(ID4)

#### 3.4.6. Availability of the Participants

Another important factor for exercise was that they had the time, as only three of the 16 participants were working. Working was not an impediment either, as they sought the necessary time because, as their physiotherapist told them, exercise had to be their priority. So, if they did not have time, they found a way. The participants agreed that the prehabilitation timetable was adequate. The sessions were held mid-morning, which was when the participants were feeling their best and had the time to come in peacefully. Since there were two sessions per week, they were able to be commit to them without overlap with other medical appointments or other personal matters. Moreover, the prehabilitation period was a time when it was easier to change habits, as all the participants emphasized that the illness had made them prioritize themselves over any other events.

*“Yes, we did the sessions in the morning, when we felt better than in the afternoon”.*

(ID8)

#### 3.4.7. Peer-Support

The most valued factor by the participants, in terms of facilitators, was the support of the group and the cohesion they felt among themselves. They mainly valued very positively the fact that they could belong to a group of women with the same type of cancer at the same stage. During the focus group, several participants highlighted that there were things that they explained to their relatives that they did not understand because they were not going through the same experience. Once in the prehabilitation group, they ended up talking about issues among themselves that they would have never talked about with their relatives for fear of making them suffer.

*“I remember the Nordic walking period with a lot of nostalgia and I get emotional”.*

(ID10)

*“We were a very close-knit group and we felt we had a lot of company”.*

(ID9)

One participant said that, although she felt the support of her family, it was not as strong as the support she felt from her prehabilitation group. Several participants stated that they have not been able to say or explain everything they needed to share with their families, in order not to make their families suffer and in order not to overly dramatize the situation they were going through.

*“Nobody can understand you better than within the group. [Other people] they couldn’t relate because they haven’t been through it”.*

(ID16)

### 3.5. Satisfaction with the Prehabilitation Program

The degree of satisfaction of the participants was very high, and they described the prehabilitation program as a great experience with important physical, psychological, and emotional benefits. Specifically, the degree of satisfaction was related to the facilities where the prehabilitation program was carried out, the material used for the sessions, and the timing of the sessions.

#### 3.5.1. Location of the Program

As mentioned earlier, easy access to outdoor facilities and the surroundings of the hospital itself were seen as facilitators of the prehabilitation program.

*“Most of us were walking and the facilities were very accessible”.*

(ID1)

Some participants would have preferred to carry out this intervention in a specific place that was not a passing area in the vicinity of the hospital and would have also liked to have lockers so that they could do their Nordic walking more calmly, without having to keep an eye on their belongings, as they did not have an area to store them.

*“Sometimes when we were exercising, we had to stop because a truck passed by and then you lost your pace”.*

(ID15)

*“There was no locker where you could leave your belongings”. *

(ID7)

#### 3.5.2. Materials Used during Prehabilitation

The participants reported that all needed equipment was provided in each session, plus that it was not only suitable for the type of exercise, but also in perfect condition.

*“Everything was provided by the physiotherapist. All we had to bring was water and our will to exercise!”*

(ID10)

#### 3.5.3. Dosage of Prehabilitation Sessions

All participants agreed that the intervention should have lasted longer, not just the two months prior to surgery. They insisted that these programs should be available not only before surgery but throughout the post-surgical process too. All participants agreed that, if these programs were an option after surgery, they would still continue to do them.

*“The exercise has to be dosed, adapted and supervised. The progression has to be assessed […]. For that, we need our physiotherapist!”*

(ID15)

With the exception of one participant who was already happy with the two sessions scheduled—because it gave her time to recover from soreness—all the others would have preferred a minimum of three sessions per week, or even more.

*“It wouldn’t have hurt to have an extra day a week […]. Maybe you missed a Monday, and then you only had one day left”.*

(ID10)

*“I would have liked it to be flexible from Monday to Friday”.*

(ID8)

All participants shared the idea that these Nordic walking and strength training sessions should be extended over a longer period, both before and after surgery. Patients in later stages of treatment could inform, encourage, and calm those at the beginning of the process. They would have appreciated being able to talk to women who had already been through it all and from whom they could learn about their experiences.

*“The program should last at least 6 months after surgery. They tell you ‘It’s all over now’ and the truth is, it’s not”.*

(ID3)

As there was no follow-up of this type of exercise nor any post-surgical rehabilitation program, right when they had the most sequelae, half of the women abandoned their daily physical activity once the program had finished. They also highlighted as very positive factors how easy learning the Nordic walking technique was and how their tolerance in terms of perceived effort had improved, which is why they felt that the program should have continued after surgery.

The participants did not feel safe exercising without a professional to guide, adapt, and supervise the exercise session at a time when they still have numerous sequelae. In fact, despite the physiotherapist providing several exercise videos to follow at home after surgery, none of the participants were consistent in doing them. Indeed, they preferred group exercise rather than doing them alone.

*“The intervention should have lasted longer, not only during the pre-surgery”.*

(ID2)

### 3.6. Guidance by the Multidisciplinary Team during Breast Cancer Treatment

This topic refers to whether the women felt accompanied by the multidisciplinary team during the process of the disease and its treatment. In the focus groups, controversy was observed in the differences among the participants’ experiences regarding this category.

#### 3.6.1. Guidance by the Multidisciplinary Team

Some participants were very grateful to the multidisciplinary team and felt that the doctors and nurses had saved their lives. They felt that both the diagnosis and treatment of the tumor was performed very quickly and effectively.

*“I can’t complain. They provided a great and very quick follow-up at all times”.*

(ID4)

*“I felt well treated and well accompanied”.*

(ID6)

One of the participants made a special mention of the fact that they felt especially supported by three professionals: the oncologist, the surgeon, and the prehabilitation physiotherapist.

*“I have been treated like a queen, but only by three professionals: the physiotherapist, the surgeon and the oncologist”.*

(ID15)

#### 3.6.2. Lack of Guidance by the Multidisciplinary Team

Many of the participants expressed that they felt very little support throughout the breast cancer process. This was mainly due to the overall lack of information on the side effects that the participants experience. In addition, they considered that there was no clear prevention of any kind about the sequelae that they were experiencing.

*“We were lucky to have prehabilitation, because otherwise we would have nothing. We were very lonely during this process”.*

(ID2)

They did not feel supported in terms of their social environment either. Participants reported that, socially, they felt frail and misunderstood, and despite many people having the best intentions, they made them feel even worse with certain unfortunate comments.

*“When I talked to other people they told me ‘It’s OK, you’ll get over it, you’re not going to die’. They have no idea”.*

(ID8)

Although treatment was initiated very rapidly, the participants considered that it is very focused on removing the tumor, but that should not be enough. They demanded solutions to several side effects that appear during and after chemoradiotherapy and surgery, as they felt like these side effects were normalized in clinical practice, with no solution to be offered.

*“I am grateful because […] they saved my life. But once you go home there is no appropriate follow-up”.*

(ID8)

Furthermore, the participants felt that there was only a medical approach to the treatment of the tumor and that there was a lack of a comprehensive approach to other unmet needs, such as psychology-related issues, appropriate nutrition, and specialized rehabilitation of the musculoskeletal system, pelvic floor, and cardiorespiratory system. For instance, after surgery, when upper limb mobility limitation, pain, oedema, etc., appeared, some participants commented that they had to wait months to be able to start rehabilitation.

*“I was in pain and they didn’t send me to rehabilitation because of the waiting list”.*

(ID8)

Participants emphasized several times during the interview that if the evidence is there for prehabilitation, then it should be accessible to all patients, not only those who take part in a clinical trial, as patients in the control group or those who do not meet the inclusion criteria cannot benefit from these programs.

*“You couldn’t come back after surgery, because the study was only for pre-surgical patients”.*

(ID8)

They also felt that there was a lack of coordination between the entire multidisciplinary team and point of contact who can refer each case to a specific specialist. Despite receiving a series of follow-up visits, in general, they experienced this process with a great deal of loneliness.

*“We all have felt very uninformed about all the consequences […]. We all go to our nurse because that’s where the oncologist sends us for information, but […] we are not informed”.*

(ID13)

Overall, the participants felt that they were accompanied in the different main areas addressed by the oncologist, the surgeon, and the physiotherapist. However, they felt that it was not enough, as they need solutions to many side effects related to BC and the treatments received. They reported feeling that their symptoms were normalized with little to no solutions.

*“They don’t provide solutions to what happens to you. They only listen to you”.*

(ID16)

#### 3.6.3. Insufficient Information on Side Effects

With regard to the information received about the side effects presented, the participants considered that very little information was provided. They would have appreciated information on the prevention of all possible side effects.

*“They hand out information in a booklet but it is very outdated and does not give answers to our side effects”.*

(ID14)

They stated that it was important to provide more information or implement group meetings where information could be exchanged with the multidisciplinary team. They also remarked that these meetings should always be face-to-face with virtual meetings only used as reinforcement.

*“We have a lot of side effects that could be prevented”.*

(ID13)

Patients were not satisfied to receive information only through leaflets which were not up to date.

*“These information leaflets only cover very basic side effects such as nausea and neuropathy, but do not go into any specifics”.*

(ID9)

The final thought of the participants is that they called for a more holistic approach to patients, with greater communication and coordination among the entire multidisciplinary team.

*“We have lacked a professional point of contact”.*

(ID3)

*“For me, nutrition, mental health, and exercise should be given the same importance during the oncological process. Because the oncologist treats a part of me, but the rest of my being... who is treating that?”*

(ID16)

## 4. Discussion

To our knowledge, this is the first qualitative study that explores perceptions and opinions of women with BC who participated in a prehabilitation program based on Nordic walking, muscle strengthening exercises, and therapeutic education with the aim of improving the future design and implementation of prehabilitation and other related programs for patients with BC.

Prehabilitation has become increasingly popular in the oncology literature as a strategy to improve the patient’s physical fitness prior to surgery, thus reducing postoperative complications [26,27,28]. Despite the scientific evidence on the effectiveness of such programs, implementation of these interventions is scarce, with reasons being mostly time-related (i.e., surgery scheduled within two weeks or less of diagnosis). However, a great proportion of patients nowadays are receiving neoadjuvant therapy prior to surgery, thus allowing for enough time and planification for prehabilitation to be implemented. Indeed, prehabilitation seems to be a feasible intervention as average adherence reported in the literature is greater than 70%. Nevertheless, the preoperative setting can be very stressful for patients having to deal with a recent cancer diagnosis, in addition to managing several medical appointments as well as other commitments. As a result, understanding patients’ preferences as well as potential facilitators and barriers to these programs is crucial for them to be successfully implemented in clinical practice [29], particularly if patients are receiving concurrent neoadjuvant therapy [30]. Our qualitative study sought to understand and analyze how patients with BC undergoing chemotherapy had experienced participation in prehabilitation and the companionship of the entire multidisciplinary team from the moment of diagnosis until after surgery.

Six main themes emerged in the interviews with the patients. The participants felt that they did not receive sufficient information to participate in the prehabilitation trial, which they identified as key issues to enhance motivation. A recent review article described the fundamental role of oncology nurses in connecting patients to these prehabilitation services. Nurses are usually the most constant and close healthcare workers, and the ones who usually perform the most follow-up visits during the entire oncology process [31]. In fact, in our study, the nurse was the person designated to perform recruitment to the trial and explain the main features of prehabilitation. However, according to patients, not much information was provided [21]. Instead, the participants agreed that it was the physiotherapist responsible for the prehabilitation who provided the most accurate information for those in the intervention group. This highlights, as seen in other studies in cancer survivors, the lack of prescription and coordination by the entire medical team—specifically by the oncologist—in the recommendation of these prehabilitation or other exercise-related programs [32,33,34]. In addition, as in other qualitative studies [35], the participants in our focus groups preferred face-to-face visits where they could be informed and prefer to be accompanied in order to obtain the greatest retention of information. Participants considered that the provided leaflets regarding the side effects of chemotherapy were outdated and did not provide a solution to their anguish when directly facing many of the side effects they were experiencing. Consistent with this, a recent clinical trial demonstrated that face-to-face education and training in preoperative abdominal physiotherapy improved patients’ ability to recall information and halved postoperative complications compared with those receiving a handout only [36]. There is no doubt that the lack of information and the difficulty in recruiting participants are among the main barriers. A systematic review of mixed methods [37] found most of the factors reported by our focus group as barriers to participation due to lack of motivation, such as lack of information about the description and benefits of prehabilitation programs, minimal recommendation by the medical team, dealing with side effects of chemotherapy, adverse weather conditions, as well as the limited time before surgery. This somehow contrasts with other studies conducted in chronic diseases such as COPD or heart failure, in which fear of exercise is one of the main barriers to participation, while according to studies performed in cancer prehabilitation, fear does not appear to be an important limitation [38,39].

All study participants felt motivated and adhered to the prehabilitation program thanks to three main factors: feeling the physical and psychological benefits of the program, sharing the experience with women with the same disease at the same stage, and the instruction and supervision of the program by a physiotherapist specializing in oncology. Therefore, these aspects were considered to be facilitators of adherence to the program, which are in line with what has been reported in other qualitative studies [19] [40]. As for the fact that the program was supervised face-to-face and took place within the hospital surroundings and not home-based, while in some studies this has been shown to be a barrier [35], the participants in this study showed it to be a facilitator, since most of the participants were off work and available, so they choose to prioritize the distraction provided by exercise and self-care to positively influence their disease over any other obligation. Patients highlighted that, despite having many medical appointments, they did not see this as a barrier to participating in the program. As shown in other studies [41], group-based and outdoor exercise are well accepted by patients not only because of its physical and psychological benefits, but also because unsupervised prehabilitation at home can sometimes be less effective due to lack of self-discipline and induce safety-issues, particularly during active cancer treatment [32,42]. Furthermore, all interviewed participants were very satisfied with the facilities and materials offered in the prehabilitation program. They showed great willingness to do supervised physical exercise because of the high level of safety and motivation they felt while doing the session. In line with our findings, in a study conducted by Ferri A. et al. on the experiences of people with cancer who had participated in a hospital-based physical exercise program, they found that most people had difficulties in maintaining exercise participation beyond the completion of a supervised hospital program [43]. Regarding the timing of these exercise programs, all participants agreed that they should be offered from diagnosis until months later throughout the recovery process in order to improve surgery-related upper limb dysfunctions and general adherence [44,45]. It is well-known that side effects from cancer treatment, both systemic and local, can endure for months and even years; thus, the participants in this study agreed that these modalities of supportive care should be offered for those who still face multiple physical and psychological problems in the long-term [30]. Therefore, improving exercise participation in cancer survivors may require supervised exercise interventions in addition to the implementation of multidisciplinary strategies to manage side effects. The goal should be to facilitate the transition from exercise to daily life, thereby improving long-term adherence [46].

On the issue of whether the participants felt supported during the illness, there is considerable controversy. As other studies have shown [47], peer-support is a key factor as it creates a significant bond which fosters great emotional benefits. While some patients interviewed felt fully supported by their healthcare team, others felt they were not and reported having received very little information on the prevention of side effects and other important issues. They felt that the medical team was very focused on the removal of the tumor, while very little emphasis was placed on the consequences of both chemotherapy and surgery. Based on the thematic analysis—and as noted in other studies [11]—our findings also highlight the potential value of including nutritional and psychological support as part of a multimodal prehabilitation program. There is a clear need to prepare patients facing this diagnosis for prolonged multimodal treatment in order to address the physical and psychological sequelae. It is important to manage their expectations by making them feel more visible to the medical team. Similarly, it is also important to identify and address issues that arise at different points in the treatment of their disease in order to improve the patients’ experience [48,49]. In this regard, the two focus groups shared ideas on ways to improve public rehabilitation services. As a result, simple initiatives emerged, such as the following: improving patient education prior to surgery, education on the benefits of exercise and encouragement of its prescription by the medical team, providing facilities for the development of multimodal (p)-rehabilitation, or coordinating multiphase programs expanding beyond the preoperative setting, accessible to the entire cancer patient population.

In summary, these results, as well as other qualitative studies in the field, highlight the need for prehabilitation programs to become more patient-centered and more accessible. This is essential in designing more effective therapeutic strategies tailored to the specific needs of patients, while overcoming barriers to effectively implement these programs [50,51].

## 5. Limitations and Strengths of the Study

This study has some limitations that need to be addressed. First and foremost, this is a substudy with patients participating in a very specific prehabilitation protocol. The population sample presented was only women and remarkably homogeneous in terms of race, ethnicity, language, and socioeconomic status, limiting the generalization of the results. Additional studies evaluating more diverse populations will likely be beneficial in identifying the unique needs of subpopulations. Most importantly, our findings are limited to the experiences of those who were randomized to the intervention arm, as we were trying to capture the insights of those participating in the sessions in order to improve the designed prehabilitation program. In the future, however, we need to incorporate, as well, the ideas and perceptions of those who might have not wanted to partake in studies such as the PREOptimize to understand their motives and improve participation in these programs. Finally, we also did not include the vision of the multidisciplinary team, which is absolutely key to effectively designing and implementing these types of interventions.

As for the strengths of the study, one important aspect is that we did not randomly select the participants for the qualitative study, but rather, we offered the possibility of participating to all women who had participated in the prehabilitation program, including those with worse adherence. In addition, to ensure that everyone had their opportunity to share ideas and observations, we constructed two focus groups, which allowed for all participants to have time to express themselves with no time-restrictions.

## 6. Conclusions

A prehabilitation program based on Nordic walking, resistance training and therapeutic education was well received among patients diagnosed with BC undergoing neoadjuvant therapy. Six main themes were identified during the focus groups, which highlighted the importance of receiving information and guidance by the healthcare team to engage in the prehabilitation program, as well as the barriers and facilitators to adhere to the intervention, including the importance of peer-support and professional supervision as main drivers to foster motivation. Future trials should include the view of patients refusing participation as well as those from the multidisciplinary team in order to effectively co-design and implement prehabilitation programs in this population.

## Data Availability

The data collected during this study are available, provided a reasonable request is made.

## Supplementary Materials

The following supporting information can be downloaded at: https://www.mdpi.com/article/10.3390/jcm13133732/s1, Table S1: Participants’ demographic information; Table S2: Participants’ clinical information.
