# Supplementary material for: Experiences of Patients with Breast Cancer Participating in a Prehabilitation Program: A Qualitative Study
Source: J Clin Med. 2024 Jun 26;13(13):3732. doi: 10.3390/jcm13133732 (PMC11242540; doi:10.3390/jcm13133732)
Supplement: Supplementary file 1 [file jcm-13-03732-s001.zip › jcm-3050619-supplementary.pdf]

Journal of
Clinical Medicine

MDPI

Table S1. Participants demographic information (both focus groups).

Participant	Sex	Age	Level of studies	Marital status	Profession	Employment Status	Cohabitation
**P-1**	Women	56	University	Single	Teacher	Sick leave	Alone
**P-2**	Women	62	Secondary	Married	Pharmacy Assistant	Sick leave	Accompanied
**P-3**	Women	57	Primary	Married	Dining Room Monitor	Sick leave	Accompanied
**P-4**	Women	58	Secondary	Married	Cleaning	Active	Accompanied
**P-5**	Women	48	Secondary	Married	Kitchen Assistant	Unemployed	Accompanied
**P-6**	Women	40	University	Single	Teacher	Active	Accompanied
**P-7**	Women	40	University	Single	Teacher	Unemployed	Accompanied
**P-8**	Women	50	Secondary	Single	Commercial	Unemployed	Accompanied
**P-9**	Women	37	University	Married	Event Organizer	Unemployed	Accompanied
**P-10**	Women	41	University	Married	Physician	Active	Accompanied
**P-11**	Women	50	University	Married	Nurse	Sick leave	Accompanied
**P-12**	Women	68	University	Separated	Teacher	Retired	Alone
**P-13**	Women	48	University	Divorced	Film director	Sick leave	Alone
**P-14**	Women	49	Secondary	Married	Catering	Sick leave	Accompanied
**P-15**	Women	41	Secondary	Married	Accountant	Sick leave	Accompanied
**P-16**	Women	50	University	Married	Teacher	Sick leave	Accompanied

Table S2. Participants clinical variables (both focus groups).

Participant	Date Diagnosis	Diagnosis	Stage	Type of surgery	Adverse effects
**P-1**	Feb-22	Luminal B	IIIA	Tumorectomy Reconstruction Lymphadenectomy	None reported
**P-2**	Des-21	Luminal B	IB	Tumorectomy Lymphadenectomy	Bacterial infection
**P-3**	Nov-21	Luminal A	IIB	Bilateral Mastectomy Lymphadenectomy	None reported
**P-4**	Aug-21	HER + Non-Luminal	IIIA	Mastectomy Reconstruction Lymphadenectomy	None reported
**P-5**	Aug-21	Basal-like (Triple Negative)	IIB	Tumorectomy Sentinel Lymph Node	Neuropathy
**P-6**	Oct-21	Luminal A	IA	Tumorectomy	Neuropathy

				Lymphadenectomy	
**P-7**	Nov-22	Basal-like (Triple Negative)	IIA	Tumorectomy Sentinel Lymph Node	Phlebitis
**P-8**	Oct-21	Luminal A	IIIA	Mastectomy Reconstruction Lymphadenectomy	None reported
**P-9**	Aug-22	HER + Non-Luminal	IIA	Tumorectomy Lymphadenectomy	None reported
**P-10**	Jul-22	Luminal A	IIA	Tumorectomy Sentinel Lymph Node	Anal fissure
**P-11**	Jul-22	Luminal A	IIA	Tumorectomy Lymphadenectomy	Lymphedema Neuropathy
**P-12**	Jul-21	Basal-like (Triple Negative)	IIIC	Tumorectomy Lymphadenectomy	Neuropathy
**P-13**	Jul-22	HER + Non-Luminal	IIA	Tumorectomy Sentinel Lymph Node	Neuropathy
**P-14**	Aug-22	Luminal A	IB	Bilateral Mastectomy Reconstruction Lymphadenectomy	Axillary Web Syndrom Neuropathy
**P-15**	Jul-22	Luminal A	IIA	Mastectomy Reconstruction Lymphadenectomy	Neuropathy
**P-16**	Set-22	Luminal A	IA	Tumorectomy Sentinel Lymph Node	Neuropathy
